# Family-level moderators of daily associations between discrimination and distress among Mexican-origin youth

**DOI:** 10.1017/S0954579424000749

**Published:** 2024-04-08

**Authors:** Kristin Valentino, Irene J. K. Park, Mario Cruz-Gonzalez, Jenny Zhen-Duan, Lijuan Wang, Tiffany Yip, Kyle Lorenzo, David Dias, Kiara Alvarez, Margarita Alegría

**Affiliations:** 1 Department of Psychology, University of Notre Dame, Notre Dame, USA,; 2 Department of Psychiatry, Indiana University School of Medicine-South Bend, South Bend, USA,; 3 Disparities Research Unit, Department of Medicine, Massachusetts General Hospital, Boston, USA,; 4 Department of Medicine, Harvard Medical School, Boston, USA,; 5 Department of Psychiatry, Harvard Medical School, Boston, USA,; 6 Department of Psychology, Fordham University, New York, USA; 7 Department of Health, Behavior and Society, Johns Hopkins Bloomberg School of Public Health, Baltimore, USA

**Keywords:** daily diary, ethnic-racial socialization, familism, family cohesion, Mexican-origin youth, racial-ethnic discrimination

## Abstract

The current study evaluated cultural values and family processes that may moderate associations between daily racial-ethnic discrimination and distress among Mexican-origin youth. Integrating micro-time (daily diary) and macro-time (longitudinal survey) research design features, we examined familism, family cohesion, and ethnic-racial socialization from youth-, mother-, and father- reports as potential buffers of daily associations between youth racial-ethnic discrimination and youth distress (negative affect and anger). The analytic sample, drawn from the Seguimos Avanzando study, included 317 Mexican-origin adolescents (M_age_ = 13.5 years) and their parents, recruited from the Midwestern United States. Results indicated that youth-reported familism and family cohesion significantly buffered daily associations between youth racial-ethnic discrimination and youth distress. In contrast, parent-reported familism and family cohesion and some aspects of ethnic-racial socialization exacerbated the discrimination to distress link. The implications of these results are discussed to inform efforts supporting the healthy development of Mexican-origin youth and their families.

## Introduction

Racial-ethnic discrimination, defined as biased and prejudiced treatment due to an individual’s race and ethnicity, plays a significant role in the development of poor health outcomes (Pascoe & Smart Richman 2009; Paradies et al., 2015). Among those most vulnerable to the risks posed by racial-ethnic discrimination are Latinx youth, who experience significantly higher rates of internalizing and externalizing symptoms compared to other ethnic groups (e.g., Alegria et al., 2019; Benner et al., 2018). This is a significant public health concern, given that the Latinx population is the largest ethnically minoritized group in the United States (Jones et al., 2021). Mexican-American adolescents report heightened levels of internalizing and externalizing symptoms, and robust research supports the adverse impact of racism and discrimination-related stressors on the mental health of Mexican-origin youth, in particular (e.g., Berkel et al., 2010, Delgado et al., 2011). Yet, although racial-ethnic discrimination has apparent adverse effects on youth mental health, there is much still to learn about the underlying mechanisms and contexts that may influence these associations, especially at the level of day-to-day experiences of Latinx youth. Taking a strengths-based approach, delineating the factors and contexts that may contribute to resilience among Mexican-origin youth is critical for informing the development of culturally tailored prevention programs. The current study adopts a family approach to examining cultural values and family-level processes that may serve as protective factors in buffering associations between daily discrimination (both personally experienced and vicarious) and distress among Mexican-origin youth.

More than two-thirds of Latinx adults and adolescents in the United States report experiencing discrimination in the last year (Zeiders et al., 2021). Whereas most research has focused on experiences of interpersonal discrimination, in which youth are directly mistreated based on their race and ethnicity, a growing body of research also now attends to vicarious discrimination, which refers to secondhand exposure to racial discrimination directed at another individual (Heard-Garris et al., 2018). Critically, vicarious discrimination is also negatively linked to youth well-being (see Heard-Garris et al., 2018, for review). As such, vicarious racism, including that directed at the family, is important to include in our conceptualization and measurement of discriminatory experiences for Latinx youth (Martin Romero & Stein, 2023).

Guided by an ecological framework and integrative theory on the development of minority children (Garcia Coll et al., 1996), as well as family-level conceptual models of racism and its impact on Latinx youth in the United States (Martin Romero & Stein, 2023), the current study evaluates potential protective factors at the family level from the perspectives of mothers, fathers, and youth that are unique to Latinx families and may shape minority youth’s mental health outcomes. As posited by the Stress Process Model (Roosa et al., 1997), cultural values play a vital role in positive development among Mexican-origin American youth. Indeed, cultural values are associated with several positive developmental outcomes for Mexican-origin youth, including prosocial behavior and reduced internalizing and externalizing psychopathology (e.g., Armenta et al., 2011; Berkel et al., 2010; Gonzales et al., 2008). Importantly, endorsement of family-related cultural values is relatively higher among first-generation families in the United States and those who are primarily Spanish-speaking (Phinney et al., 2000), making cultural values salient among Mexican-origin youth living in new migration areas such as the Midwest. Indeed the South and Midwest are the largest growth areas for Latinx immigration in the United States (e.g., Crowley et al., 2015; Jacques et al., 2019). In Indiana, the setting of the current study, the Latinx population doubled between 2000 and 2010 and increased by another 40% between 2010 and 2020 to reach approximately 8.2% of the state’s population (US Census Bureau, 2023).

Several cultural values and family processes may serve as buffers of the link between discrimination and mental health among Mexican-origin families (Martin Romero & Stein, 2023). In particular, the family is highly valued within Mexican culture. *Familism* is a cultural value that emphasizes family interdependence, loyalty, and a sense of duty and responsibility to care for one another (Behnke et al., 2008; Gaines et al., 1997). Relatedly, the centrality and importance of close-knit family relationships in Mexican culture are reflected in the construct of family cohesion. *Family cohesion* refers to family members’ emotional bonding towards each other (Olson et al., 1979). Finally, *ethnic-racial socialization*, the process through which parents teach youth about their cultural heritage and practices, as well as how to cope with racism as ethnically minoritized members of a racist society (Huynh & Fuligni, 2008), is a common practice among Mexican-origin families. It aims to support adaptive development in the context of discrimination (e.g., Umaña-Taylor & Fine, 2004). Below, we elaborate on these family-level constructs and their connection to mental health among Mexican-origin youth. By identifying effective family processes that may help youth cope with the adverse effects of discrimination at the daily level, we seek to directly inform prevention efforts to reduce the impact of racial-ethnic discrimination on mental health among Mexican-origin adolescents.

### Family-level protective factors

Familism is a core cultural value featured in conceptual models of child development & parenting among Latinx families (Cahill et al., 2021; Calzada et al., 2012). Familism refers to the emphasis in traditional Mexican culture on family interdependence, the responsibility to care for each other, and the responsibility of family members to consider the needs and desires of the family when making decisions for themselves (Sabogal et al., 1987). Familism values are expected to be protective by ensuring the family remains a central influence on youth identity development, thereby acting as a buffer against external assaults on identity, such as racial-ethnic discrimination. Prior research evidences that familism may operate as a buffer, discouraging engagement in problematic behaviors and fostering prosocial tendencies both within and outside the family (Berkel et al., 2010; Gonzalez et al., 2008). Among Latinx adolescents, familism values have been directly and indirectly associated with multiple forms of prosocial behavior (e.g., Knight et al., 2014; Streit et al., 2020) as well as linked to lower externalizing behaviors (Gonzales et al., 2008; Marsiglia et al., 2009). Indeed, a recent comprehensive meta-analysis of familism values among Latinx individuals across the lifespan found significant positive direct associations between familism and family warmth and support and negative associations between familism and individual internalizing, externalizing, and family conflict (Cahill et al., 2021). However, the strength of some of these associations is moderated by the youth developmental period and the measure of familism included. For example, the association between familism and internalizing is stronger among youth in early compared to later adolescence, and when assessed with familism measures that include items focusing more heavily on family emotional support (Cahill et al., 2021).

There is also evidence that familism is protective in reducing the effects of adverse experiences on youth mental health. For example, familism buffers the link between deviant peer relationships and externalizing symptoms in Mexican-origin youth (German et al., 2009), as well as links between material stress and depression (Montoro & Ceballo, 2021). However, other studies have found mixed evidence regarding the role of familism in associations between discrimination and Mexican-origin adolescent adjustment (e.g., Umaña-Taylor et al., 2011; Stein et al., 2015), with maternal endorsement of familism, at times, exacerbating risk for externalizing behavior among Mexican-origin females who experienced discrimination (Delgado et al., 2011). In explaining this exacerbating role of maternal familism, Delgado and colleagues suggested that when youth react to experiences of discrimination with distress, mothers may demonstrate high levels of familism to try to steer children back towards the family for support. However, relatively little is known about the potential impact of familism values as a protective factor for managing daily-level responses to stressors such as discrimination over time. Emerging evidence suggests that familism may promote more adaptive responses to daily stress (Santiago et al., 2016). Given familism’s central role as a core cultural value among Mexican-origin families, understanding its potential role in moderating associations between daily experiences of discrimination and youth distress is an essential goal of the current study. Because youth begin to internalize cultural values during adolescence (Knight et al., 2011), familism values may be important for youth emotional adjustment. Moreover, as youth cope with experiences of vicarious discrimination towards others, including family members, each parent’s unique familism values may be necessary for understanding the impact of daily racial-ethnic discrimination on youth distress.

Family cohesion is another critical family process related to the centrality of the family, valued among Mexican-origin families. Different from familism, which is a cultural value, family cohesion refers to the emotional connection and closeness between family members. Higher endorsement of family cohesion is reported among Mexican-origin families compared to non-Latinx White families (Baer & Schmitz, 2007). Greater cohesion between family members appears to be a source of support that helps Latinx families face mental health challenges, though findings have been mixed. For example, family cohesion is negatively related to trauma symptoms (Singh et al., 2011). Specific to adolescents, family cohesion is protective against rule-breaking and conduct problems (Marsiglia et al., 2009) and is positively associated with Latinx adolescents’ psychological well-being (Lorenzo-Blanco et al., 2012). Similarly, in Latinx samples, family cohesion is negatively related to psychological distress (Rivera et al., 2008).

Limited research has focused on the role of family cohesion in affecting adolescent well-being at the daily level. In a majority non-Latinx White sample, family cohesion was positively associated with daily well-being (Fosco & Lydon- Staley, 2019). Moreover, at the within-family level, on days when family cohesion was higher than usual, adolescents felt less depressed, anxious, and angry and they had higher positive mood, life satisfaction, and meaning and purpose in life (Fosco & Lydon- Staley, 2019). Additional research within Mexican-origin families is needed to further address the potential role of family cohesion as a protective family process that may buffer associations between daily experiences of discrimination and youth distress.

Ethnic-racial socialization is the process through which ethnically and racially minoritized parents aim to instill cultural heritage and values in their children and prepare them to navigate the challenges of discrimination associated with their ethnicity and race (Ayon et al., 2020; Huynh & Fuligni, 2008). Existing reviews of ethnic-racial socialization have identified four primary aspects of ethnic-racial socialization: cultural socialization, preparation for bias, promotion of mistrust, and egalitarianism (Hughes et al., 2006; Umaña-Taylor & Hill, 2020; Wang et al., 2020). Cultural socialization refers to positive messages about an in-group’s culture and history to facilitate the youth’s ethnic pride. Preparation for bias refers to how parents attempt to enhance youth awareness of discrimination and cope with it. Promotion of mistrust refers to parent efforts to emphasize the need for wariness or distrust of the out-group in inter-racial interactions. Finally, egalitarianism messages emphasize equality among ethnic-racial groups (Hughes et al., 2006). Research with Latinx families specifically, indicates that Latinx families engage in each of these forms of ethnic-racial socialization, which are consequential for youth outcomes including ethnic-identify development, academic adjustment, mental and behavioral health (Ayon et al., 2020). Further, parents’ documentation status influences their ethnic-racial socialization, with undocumented Latinx parents engaging in more cultural socialization and preparation for bias than Latinx parents who are documented (Cross et al., 2020). Ethnic-racial socialization may be especially relevant in new migration areas like Indiana, where approximately 30 % of immigrants are undocumented (American Immigration Council, 2020).

Perhaps through developing a sense of cultural pride, affiliation, and positive regard for one’s culture, cultural socialization has been associated with positive psychosocial and behavioral outcomes for youth of color (Umaña-Taylor & Hill, 2020; Wang et al., 2020) and for Mexican-origin adolescents specifically (Ayón et al., 2020; Huyhn & Fuligni, 2008). Conversely, promoting mistrust has been associated with poor youth outcomes, including worse emotional adjustment (Dunbar et al., 2015; Huynh & Fuligni; Liu & Lau, 2013). Especially relevant to the current analyses, cultural socialization and preparation for bias have buffered associations between discrimination and maladjustment among racial and ethnic minority youth, although findings have been mixed (for reviews, see Ayon et al., 2020, Jones & Neblett, 2017, Umaña-Taylor & Hill, 2020). For example, parents’ promotion of trust has been associated with adolescent depressive symptoms among undocumented Latinx families (Cross et al., 2020). Preparation for bias, mainly when practiced by fathers, has been shown to exacerbate associations between discrimination and adolescent mental health among Mexican-origin youth (Park et al., 2020). Consistent with this literature, we aimed to evaluate the potential moderating roles of cultural socialization, preparation for bias, and promotion of mistrust in the association between daily experiences of discrimination and youth distress. Moreover, we aimed to advance research on how ethnic-racial socialization practices operate within Mexican-origin families by expanding beyond single reporters and assessing ethnic-racial socialization from the perspective of youth, mothers, and fathers.

Adolescence is a critical developmental period when youths begin the process of racial and ethnic identity exploration and commitment and internalize cultural values (e.g., Knight et al., 2011; Tatum, 2017; Yip et al., 2019). Assaults to their racial and ethnic identity in the form of racial and ethnic discrimination may be especially detrimental during this time, and protective family processes may be especially beneficial. Parents are more likely to explicitly discuss the topics of race and ethnicity with their children once they reach adolescence (e.g., Tatum, 2017). Similarly, adolescents may be ready to engage in discussions of racism as they accrue more autonomy and experience racism in settings outside of the home and without their family present (Neblett et al., 2008). As such, adolescence is a critical time for evaluating the influence of cultural values and family processes on how children cope with experiences of discrimination.

### Current study

Although there are strong conceptual and empirical reasons to anticipate that familism, family cohesion, and ethnic-racial socialization may moderate the adverse effects of discrimination on youth distress and mental health, much of the literature in this area has focused on between-person differences in discrimination in association with mental health. In contrast, the current study focuses on a design that combines a between- and within-persons design. While certainly informative, between-person approaches have some limitations, especially when examining the effects of discrimination. Between-person differences in self-reported discrimination are conceptualized as differences in exposure to discriminatory events. However, between-person differences in other characteristics, such as personality or identity, may influence how everyday unfair treatment is perceived and reported as discrimination on questionnaire measures (McCrae, 1990). A within-person design addresses limitations by using each person as its own control (Hoffman & Stawski, 2009). Using longitudinal data, Park and colleagues parsed out within- and between-person effects and established within-person associations between Mexican-origin youth’s experiences of discrimination with anger and internalizing symptoms (Park et al., 2017). In the current study, we build on this important work using daily dairy methods to investigate daily-level linkages between discrimination and affect, as others have done (e.g., Huynh & Fuligni, 2010; Potter et al., 2019; Torres & Ong, 2010). Assessment of daily-level associations allows for examination of dynamic within-person day-to-day impacts of stress exposure (Ohly et al., 2010). Thus, at the daily level, we assessed youth experiences of discrimination, anger, and negative affect as a daily proxy for internalizing symptoms.

As such, the current study makes several novel contributions to the literature. First, we integrate both micro-time (daily diary) and macro-time (baseline assessment from a longitudinal survey) research design features, enabling us to capture daily fluctuations within individuals and differences between individuals. This methodological innovation advances the current science on health disparities by allowing us to understand how moderating processes influence daily-level adolescent responses to discrimination. Moreover, we evaluate family-level moderators of the discrimination to distress link among youth, including a comprehensive assessment of youth discrimination that includes interpersonal and vicarious experiences and a broad assessment of family-level processes from the perspectives of youth, mothers, and fathers. This design provides an opportunity to inform essential family practices that may be cultivated to support Latinx youth to cope with discrimination and to promote mental health, with direct potential application to developing new culturally-informed prevention programs. Finally, the inclusion of data from youth, mothers, and fathers is a valuable contribution to the literature, given the absence of studies on Latinx fathers (Cabrera & Bradley, 2012) as well as the unique contributions of fathers to mental health among Mexican-origin youth (e.g., Park et al., 2020). In this study, all three family members provided reports of the family-level protective factors tested as moderators of the daily association between youth-reported discrimination and distress.

### Hypotheses

Our fundamental hypothesis was that the adverse effects of discrimination on youth distress at the daily level (micro-time) would be moderated by protective factors at the family level (using macro-time variables). In particular, we hypothesized that familism and family cohesion would buffer the link between daily discrimination to distress. Among ethnic-racial socialization processes, we hypothesized that greater cultural socialization and preparation for bias would be protective. In contrast, greater promotion of mistrust may exacerbate risk, especially for fathers, as observed by Park et al., 2020. In general, we anticipated that the moderation patterns would be consistent across the three reporters of family-level protective factors (youth, mother, father). Still, father-reported ethnic-racial socialization factors may be especially critical influences on youth mental health among Mexican-origin families, given prior research findings that fathers’ (but not mothers’) discrimination experiences and promotion of mistrust exacerbate the association between discrimination and distress among their adolescent children (Park et al, 2018; 2020).

## Method

Data come from the *Seguimos Avanzando* study, a longitudinal study of Mexican-origin youth and their parents living in Northern Indiana, aimed at understanding the key mediating and moderating mechanisms that affect the link between discrimination-related stressors and mental health outcomes for Latinx youth (Alegria et al., 2024). Northern Indiana was selected as a region of interest to examine the discrimination-related stressor-mental health link among Mexican-origin youth living in a new migration area. The Latinx population in Indiana increased by 140% between 2000 and 2020, and is now estimated to be approximately 8% of the state population (US Census Bureau, 2023). Data for this study come from the Wave 1 assessment and the 21-day daily diary burst, which immediately followed Wave 1.

### Participants

Utilizing an ethnic-homogeneous design, we recruited 344 Mexican-origin families, including adolescents aged 12–15, with at least one parent between April 2021 and December 2022. We selected this age range to observe developmental changes in transitioning into and through adolescence. We aimed to enroll mothers and fathers whenever possible. However, to obtain a representative sample of Mexican-origin families in our region, we did not exclude single-parent families. We enrolled 335 mothers and 171 fathers in total. Inclusion criteria were: (1) the family has an adolescent, age 12–15 years old, of Mexican descent; (2) both the adolescent’s biological parents are of Mexican origin; (3) the adolescent is residing with either a biological parent or a legal guardian, also of Mexican origin. The exclusion criteria were: (1) if the parent reported the adolescent had a severe learning or developmental disability, which would prevent understanding of survey responses, or (2) if the family already participated in our prior Adelante study of Mexican-origin families (Park et al., 2017). In the present study, *n* = 27 adolescents who did not participate in the daily diary burst were excluded. This resulted in a final analytical sample of 317 adolescents, 309 mothers, and 164 fathers. These 317 adolescents had a mean age of 13.5 years old (SD = 1.1), 50.8% self-reported their gender as male (*n* = 161), 46.7% as female (*n* = 148), and 2.5% as non-binary or third gender (*n* = 8). Mothers had a mean age of 41.2 years old (SD = 6.2), 62.5% were married (*n* = 193), 23.0% were unmarried (*n* = 71), 14.5% were separated (*n* = 45), and reported a median yearly income between $20K and $29K. Fathers had a mean age of 44.1 years old (SD = 7.9), 81.7% were married (*n* = 134), 14.0% were unmarried (*n* = 23), 4.3% were separated (*n* = 7), and reported a median yearly income between $40K and $49K. The vast majority of youth were born in the United States (93.7%), with the remainder born in Mexico. In contrast, nearly all parents were born in Mexico (92.6% of mothers, 94.3% of fathers).

### Recruitment

Recruitment procedures followed best practice recommendations for recruiting and retaining Latinx immigrant families (Martinez et al., 2012). A trained bilingual and bicultural staff cultivated strong community partnerships and obtained support from several key institutions and organizations serving the Latinx community in Northern Indiana. In particular, recruitment efforts focused on in-person, individual recruitment at churches, health clinics, schools, and community organizations serving Latinx families in the area.

### Procedures & design

The study includes three waves of longitudinal data collection for youth, mothers, and fathers, spaced approximately 9–12 months apart. In addition, the youth completed a 21-day daily diary burst after completing the Wave 1 assessment. Families were invited to conduct their interviews at our research lab, in their homes, or at one of several community sites (e.g., local health clinic, church, public library) or to complete their interview via Zoom to facilitate participation. We used a multi-step process of translation and adaptation designed to achieve semantic, content, and technical equivalence, including translation, back-translation, and bilingual expert review, to translate any measures not currently available in Spanish. We also pilot-tested the study survey and conducted psychometric analyses to assess measurement invariance across languages (Alegria et al., 2024).

### Measures

#### Baseline (Wave 1) interview

Trained bilingual staff obtained written parental consent for themselves and their children’s participation in the study and youth assent. Participants could consent and complete interviews in their preferred language (English or Spanish). Most parents (91.0% of mothers and 91.8% of fathers) chose to be interviewed in Spanish, whereas most youth (96.2%) opted to complete their assessment in English. Baseline interviews included reports of the moderators of interest in familism, family cohesion, and ethnic-racial socialization from all family members (youth, mother, father).

#### Moderators from wave 1

##### Familism

Familism was measured using a modified 10-item scale developed initially by Sabogal and colleagues (Sabogal et al., 1987). This adapted scale has been used in prior work with Latinx youth and families and has strong psychometric properties (Bird et al., 2006). Participants rated aspects of familism, including familial obligation, support from family, and family as referents, using a 5-point scale ranging from 1 (Very Much in Disagreement) to 5 (Very Much in Agreement). In this sample, internal consistency (Cronbach’s α) was adequate for all reporters (youth α = 0.70; mother α = 0.75; father α = 0.76).

##### Family Cohesion

Family Cohesion was assessed using the FACES-IV, a scale developed to evaluate family dynamics (FACES-IV; Olson, 2011). The cohesion subscale comprises ten items and asks respondents about the emotional bonding among family members. Participants indicate how frequently behaviors occur in their family (e.g., “In our family, we like to spend our free time together”) on a scale ranging from 1 (almost never) to 5 (almost always). Internal consistency was high (youth α = 0.90; mother α = 0.75, father α = 0.80).

##### Ethnic-Racial Socialization

Ethnic-Racial Socialization was assessed with the Ethnic Socialization Scale (Hughes & Chen, 1997). This 13-item scale assesses three areas of ethnic-racial socialization in three subscales: cultural socialization (5 items), preparation for bias (6 items), and promotion of mistrust (2 items). Mothers and fathers reported how many times in the past year they talked with their adolescents about various issues related to ethnic socialization on a 5-point scale, ranging from 1 (never) to 5 (six or more times). Similarly, youth reported how often they discussed these things with their parents in the past year. Mean scores were computed for the three subscales. Internal consistency for each scale was adequate: cultural socialization, youth α = 0.77, mother α = 0.81, father α = 0.85; preparation for bias, youth α = 0.82, mother α = 0.82, father α = 0.82; promotion of mistrust, youth α = 0.66, mother α = 0.60, father α = 0.62. Although the alphas for promoting mistrust are relatively low, they are consistent with other published studies using this subscale (e.g., Park et al., 2020, mother α = 0.38, father α = 0.50).

#### Daily diary

Following the baseline assessment, the youth were invited to participate in a 21-day daily diary, examining mechanisms in the daily link between discrimination-related stressors, distress, and sleep. As in other daily diary studies of discrimination (e.g., Yip et al., 2020) youth completed the surveys daily before bed (once/day) for three weeks (21 days). Youth received a daily link to the 5–10-minute assessment, hosted on Qualtrics, on their phone or other personal device. Tablets were provided to those who did not have access to a device. Youth were compensated for each day of daily diary completion, with bonuses added for each complete week of participation. Research assistants monitored compliance and contacted youth to problem solve after two consecutive missed days to facilitate participation. Youth were compliant with their daily diary completion (mean [SD] = 14.27 [6.89] diaries completed, median = 17 days). Daily diary interviews were used to obtain youth reports of the focal predictor of racial-ethnic discrimination and the distress outcomes of negative affect and anger.

#### Focal predictor from daily diary

##### Daily Racial-ethnic discrimination.

Adolescents’ daily racial-ethnic discrimination was measured using a four-item scale that assessed exposure to direct, online, or vicarious racial-ethnic discrimination. The first item (“Today, others treated me poorly because of my race/ethnicity”), was selected from the Racial/Ethnic Discrimination Index (REDI; Feng et al., 2021) as recommended by the authors, as the item has the highest information, according to IRT analyses. Three additional items were variations of the first item, reflecting different referents: “Today, others treated my friends/family/peers poorly because of their race/ethnicity,” “Today, others treated me poorly online because of my race/ethnicity,” and “Today, other people were treated poorly because of their race/ethnicity. The scale was rated on a 3-point Likert scale ranging from 1 (did not happen/was not a problem today) to 3 (very much a problem), with higher scores reflecting more ethnic-race-based discrimination. The scale has demonstrated good internal consistency at the daily level (α = 0.90) and criterion-related validity (Feng et al., 2021). In the current sample, we observed adequate internal consistency at the daily level (α = 0.69) and youth level (α = 0.73). The intraclass correlation coefficient (ICC) for daily racial-ethnic discrimination was 0.44, reflecting considerable variation across days across participants. As shown in Table 1, on average, youth were more likely to report exposure to any ethnic-racial discrimination at the beginning of the 21-day daily diary survey (% of youth reporting “Somewhat of a problem today” or “Very much of a problem today”). Further, youth were also more likely to report exposure to vicarious ethnic-racial discrimination than direct or online racial-ethnic discrimination. In multilevel exploratory factor analysis, we found that only one factor could be fitted to all four items both at the between-youth and at the within-youth levels. This one factor model had a very good fit in our data: χ^2^(2) = 7.32, *p* = 0.12; CFI = 0.998; TLI = 0.995; RMSEA = 0.013. Therefore, all four items were summed to create a composite discrimination score, which was highly correlated with both interpersonal (direct or online, *r*-between = 0.84, *r*-within = 0.73) and vicarious ethnic-racial discrimination (*r*-between = 0.96, *r*-within = 0.86).

#### Youth distress outcomes from daily diary

##### Daily negative affect.

The International Positive and Negative Affect Scale, Short Form (PANAS-SF; Thompson, 2007) assessed youth’s positive and negative affect. This 10-item short form of the original PANAS scale (Watson et al., 1988) has demonstrated strong psychometric properties across several international samples of different cultural backgrounds. The cross-sample stability, internal reliability, temporal stability, cross-cultural factorial invariance, and convergent and criterion-related validities of the PANAS-SF were found to be psychometrically acceptable. In the current study, we focused on the negative affect scale (i.e., afraid, nervous, upset, hostile and ashamed), which had adequate internal consistency at the daily level (α = 0.71) and the youth level (α = 0.83). The ICC for negative affect was 0.64.

##### Daily anger.

The NIH Toolbox-Anger Scale, NIHTB-A (Pilkonis et al., 2013), a modified version of the Anger Affect Fixed Form Ages 8–17 v2.0 from the NIH Toolbox for Assessment of Neurological and Behavioral Function, was used to assess youths’ daily experiences of anger. The measure was adapted by adding the timeframe of “today” to items. For example, “Today, I felt mad” and “Today, I was so angry I felt like yelling at somebody” on a 5-point scale ranging from 1 (never) to 5 (almost always). The internal consistency of the NIHTB-A in our study sample was strong (daily-level α = 0.83; youth-level α = 0.86). The ICC was 0.53.

### Data analytic strategy

We estimated the moderating effects of familism, family cohesion, and ethnic-racial socialization in the relationship between youth racial-ethnic discrimination and youth distress within a multilevel modeling (MLM) framework. MLM was employed given the hierarchically clustered structure of our data, in which days at Level-1 (*i* = 1, …, *n*_*j*_; *n*_*j*_ ∈ [0, 1, …, 21 *days*]) were clustered within youth at Level-2 (*j* = 1 … *J*). Expressly, we were interested in testing whether the effect of youth racial-ethnic discrimination (*x*_*ij*_, the Level-1 focal predictor measured daily) on youth distress (*y*_*ij*_, the Level-1 outcome measured daily) varied across levels of youth-, mother-, and father-reported familism, family cohesion, and ethnic-racial socialization (*z*_*j*_, the Level-2 moderator that does not vary within youth). Following the notation of Preacher et al. (2016), we denote this as a 2 × (1→1) design, where “2” is the level at which the moderator is measured, the first “1” is the level at which the focal predictor is measured, and the second “1” is the level at which the outcome is measured.

In MLM, Level-1 variables can be decomposed into two components: one that varies only *between* Level-2 units (in our case *between-youth*), and one that varies only *within* Level-2 units (in our case *within-youth*) (Raudenbush & Bryk 2002; Snijders & Bosker, 2012). For the focal predictor (*x*_*ij*_, youth racial-ethnic discrimination), the *between-youth* component was captured using cluster means (i.e., average across days for each youth), which we denote *x*_·*j*_. The *within-youth* component was captured using scores centered around the cluster means, which we denote as *x*_*i*_ = *x*_*ij*_ − *x*_·*j*_ (Wang & Maxwell, 2015). For our focal predictor, the *between-youth* effect of *x*_·*j*_ measures whether youth who report more racial-ethnic discrimination differ in their reports of distress compared to youth who report less racial-ethnic discrimination. The *within-youth* effect of $x_{ij}^{*}$ measures whether a youth reports more or less distress on days when he or she reports higher or lower discrimination. Level-2 variables, which are measured at the cluster level, do not have a *within* component because they do not vary within Level-2, and are thus regarded as between variables only. Accordingly, Level-2 variables can have a between-person effect or a cross-level effect.

Since Level-1 variables can have between and within effects, moderation can occur at the between-level (interaction of two Level-2 variables), at the within-level (interaction of two Level-1 variables), and at the cross-level (interaction of a Level-1 variable and a Level-2 variable). In our context, the focal predictor of racial-ethnic discrimination, *x*_*ij*_, is a Level-1 variable that can have *between-youth* (*x*_·*j*_) and *within-youth* effects $\left(x_{ij}^{*}\right)$ on youth distress, *y*_*ij*_. However, the moderators, *z*_*j*_, are Level-2 variables that can only have a *between-youth* effect or a *cross-level* effect. Thus, moderation effect of *z*_*j*_ can occur at the between-level (*x*_·*j*_ × *z*_*j*_) or at the cross-level $\left(x_{ij}^{*}\times z_{j}\right)$. Our multilevel moderation model is represented by the following system of equations:

$$\text{Level-1: }y_{ij}=\beta_{0j}+\beta_{1j}x_{ij}^{*}+\beta_{2j}{\text{day}}_{ij}+\beta_{3j}{\text{weekend}}_{ij}+\varepsilon_{ij}$$

$$\text{Level-2: }\beta_{0j}=\gamma_{00}+\gamma_{01}x_{\cdot j}+\gamma_{02}z_{j}+\gamma_{03}\left(x_{\cdot j}\times z_{j}\right)+w_{j}+u_{0j}\;\beta_{1j}=\gamma_{10}+\gamma_{11}z_{j}+X_{j}+u_{1j}\;\beta_{2j}=\gamma_{20}\;\beta_{3j}=\gamma_{30}$$

where *y*_*ij*_, $x_{ij}^{*}$, *x*_·*j*_, and *z*_*j*_ are defined as above. Since testing effects can arise due to repeated administration of the same measures, day of daily diary interview (*day*_*ij*_) was included as a Level-1 covariate to adjust for the linear effect of time (Wang & Maxwell, 2015). Further, we observed that youth were less likely to complete the daily diary interviews on Fridays and Saturdays. Thus, we also adjusted for a weekend effect, *weekend*_*ij*_, coded Friday or Saturday as 1 and other days as 0.

As shown elsewhere (Alegria et al., 2024), analysis of missing data at both the *between*- and *within-youth* levels indicated that families with no missing data (i.e., families where youth, mother, and father all participated) did not statistically differ from families with some missing data (*n* = 9 families where the mother did not participate and *n* = 173 families where the father did not participate). The few exceptions were mother-reported marital status (mothers in families with no missing data were more likely to report being married at Wave 1), youth-reported gender (females completed more days from the daily diary), and youth survey mode (those who responded to the Wave 1 interview in person completed more days from the daily diary). Thus, we adjusted for the Level-2 vector of Wave 1 covariates *w*_*j*_, which included youth age (12–15 years old), gender (male versus nonmale), mother-reported marital status (married versus unmarried), and youth Wave 1 survey mode (in person versus other). The composite-form of the model is as follows:

$$y_{ij}=\gamma_{00}+\gamma_{01}x_{\cdot j}+\gamma_{02}z_{j}+\gamma_{03}\left(x_{\cdot j}\times z_{j}\right)+\Gamma_{0}^{\prime }w_{j}+\gamma_{10}x_{ij}^{*}\;+\gamma_{11}\left(x_{ij}^{*}\times z_{j}\right)+\Gamma_{1}^{\prime }\left(w_{j}\times x_{ij}^{*}\right)+\gamma_{20}{\text{day}}_{ij}+\gamma_{30}{\text{weekend}}_{ij}\;+u_{0j}+u_{0j}x_{ij}^{*}+\varepsilon_{ij}$$

In the above equation, there are two moderation effects of interest: The between-level interaction (*x*_·*j*_ × *z*_*j*_) with fixed effect *γ*_03_ and the cross-level interaction $\left(x_{ij}^{*}\times z_{j}\right)$ with fixed effect *γ*_11_. *γ*_03_ measures whether the *between-youth* effect of racial-ethnic discrimination on youth distress is moderated by overall youth levels of the moderator. Similarly, *γ*_11_ measures whether the *within-youth* effect of racial-ethnic discrimination on youth distress is moderated by overall youth levels of the moderator. Using youth-reported family cohesion as an example, the between-level interaction captures the extent to which, conditional on reporting the same average level of racial-ethnic discrimination, a youth with high levels of family cohesion reports lower or higher average distress scores compared to a youth with low levels of family cohesion. The cross-level interaction captures the extent to which the within-person association between racial-ethnic discrimination and youth distress is stronger or weaker for a youth with high family cohesion compared to a youth with low family cohesion. All analyses were conducted in the Stata software version 17 (StataCorp, 2021) and in the RStudio software 2023.3.1.446 (Posit team, 2023). As recommended within a MLM framework, missing data was handled using model-based multiple imputation using Blimp 3.0 (Enders et al., 2020), under the plausible assumption that conditional on the observed covariates, the missingness mechanism was missing at random.

## Results

### Descriptive statistics

As shown in Table 2, at the *between-youth* level, youth-reported racial-ethnic discrimination, negative affect, and anger were all positively and significantly correlated with each other. Further, youth-reported racial-ethnic discrimination was significantly negatively correlated with youth-reported family cohesion (*r* = −0.18) and positively correlated with youth- and mother-reported preparation for bias (*r* = 0.19 and *r* = 0.12, respectively) and youth-reported promotion of mistrust (*r* = 0.14). Youth-reported negative affect was significantly negatively correlated with youth-reported family cohesion (*r* = −0.12) and positively correlated with youth-reported preparation for bias (*r* = 0.14) and promotion of mistrust (*r* = 0.15). Youth-reported anger was significantly negatively correlated with their own reports of familism and family cohesion (*r* = −0.12 and *r* = −0.15, respectively).

Regarding youth, mother, and father reports of the moderators, youth-reported familism was not significantly correlated with the mother or father report. Youth reports of family cohesion were significantly correlated with both mother (*r* = 0.15) and father reports (*r* = 0.28). Youth-reported cultural socialization and preparation for bias were significantly positively correlated with mother (*r* = 0.15 and *r* = 0.16, respectively) and father reports (*r* = 0.25 and *r* = 0.17, respectively). Finally, youth-reported promotion of mistrust was not significantly correlated with the mother report but was significantly positively correlated with the father report (*r* = 0.23).

### Negative affect

**Figure 1.**
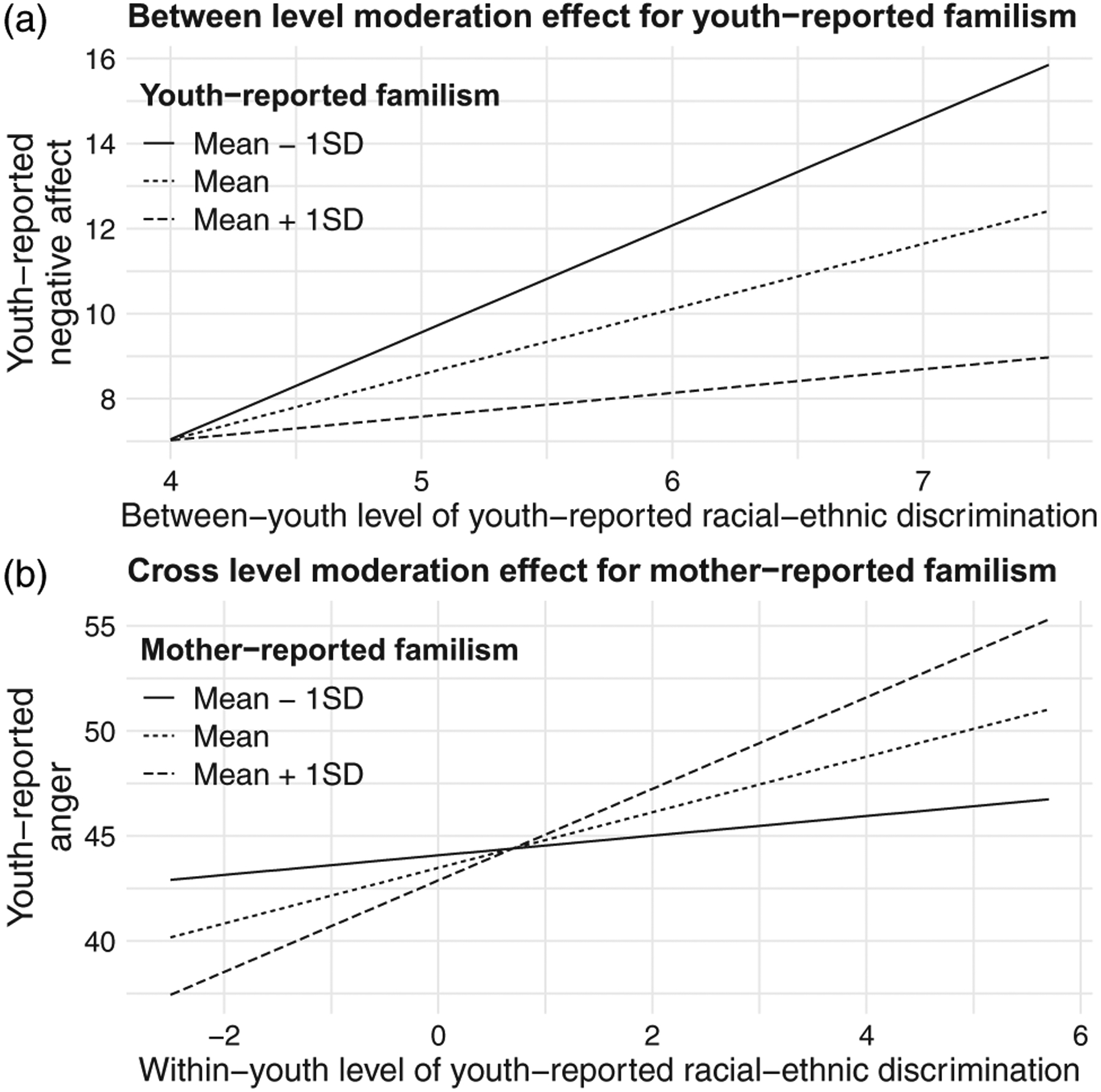
An example of between-level and cross-level significant moderation effects. Notes: SD, standard deviation.

The moderating effects of youth-, mother-, and father-reported familism, family cohesion, and ethnic-racial socialization in the relationship between daily youth-reported exposure to racial-ethnic discrimination and daily youth-reported negative affect are presented in Table 3. At the between-level, we found that familism (youth and father report), family cohesion (youth and mother report), cultural socialization (father report), and preparation for bias (father report) significantly moderated the relationship between youth racial-ethnic discrimination and youth negative affect (coefficient *γ*_03_). As outlined above, the *between-youth* effect measures whether youth who report more racial-ethnic discrimination differ in their reports of negative affect than youth who report less racial-ethnic discrimination. The significant between-level moderation effect for youth self-reported familism and family cohesion suggests that the adverse *between-youth* effect of racial-ethnic discrimination on negative affect was weaker for youth with higher levels of self-reported familism and family cohesion compared to youth with lower levels of self-reported familism and family cohesion (*γ*_03_ = −0.26, 95% CI = [−0.45, −0.06] and *γ*_03_ = −0.09, 95% CI = [−0.18, −0.01], respectively), which was consistent with our hypotheses. In Panel A of Figure 1, we provide a graphical representation of the significant between-level moderation effect for youth self-reported familism. To do so, we plotted the *between-youth* effect of racial-ethnic discrimination on youth negative affect for different values of youth-reported familism: low (1 SD below the mean), average, and high (1 SD above the mean). Consistent with our coefficient estimates, Panel A of Figure 1 shows that at every *between-youth* average level of racial-ethnic discrimination, youth with high levels of self-reported familism (1 SD above the mean) had lower average levels of negative affect compared to youth with low levels of self-reported familism (1 SD below the mean).

In contrast, the moderating effects of father-reported familism, mother-reported family cohesion, father-reported cultural socialization, and father-reported preparation for bias acted in the opposite direction. Regarding father-reported familism, our results indicated that the adverse *between-youth* effect of racial-ethnic discrimination on negative affect was stronger for youth with higher levels of father-reported familism compared to youth with lower levels of father-reported cultural socialization (*γ*_03_ = 0.23, 95% CI = [0.08, 0.38]). A similar result was observed for mother-reported family cohesion, father-reported cultural socialization, and father-reported preparation for bias. Specifically, the adverse *between-youth* effect of racial-ethnic discrimination on negative affect was stronger for youth with higher levels of mother-reported family cohesion, father-reported cultural socialization, and father-reported preparation for bias compared to youth with lower levels (*γ*_03_ = 0.13, 95% CI = [0.01, 0.24]; *γ*_03_ = 0.83; 95% CI = [0.09, 1.57]; and *γ*_03_ = 1.23, 95% CI = [0.28, 2.18], respectively). These moderating effects were opposite to those predicted by our hypotheses.

At the cross-level, no significant moderation effects were observed for either youth, mother, or father reports of the moderators (coefficient *γ*_11_). That is, the *within-youth* effect, which measures the extent to which a youth reports higher or lower levels of negative affect on days when he or she reports higher or lower levels of exposure to discrimination, did not vary across levels of youth-, mother-, and father-reported familism, family cohesion, and ethnic-racial socialization.

### Anger

The moderating effects of youth-, mother-, and father-reported familism, family cohesion, and ethnic-racial socialization in the relationship between youth racial-ethnic discrimination and youth anger are presented in Table 4. At the between-level, we found that youth- and father-reported familism significantly moderated the association between youth racial-ethnic discrimination and youth anger. Expressly, the adverse *between-youth* effect of racial-ethnic discrimination on anger was weaker for youth with higher levels of self-reported familism compared to youth with lower levels of self-reported familism (*γ*_03_ = −0.62, 95% CI = [−1.18, −0.06]), consistent with our hypotheses. In contrast, our results indicated that the adverse *between-youth* effect of racial-ethnic discrimination on anger was stronger for youth with higher levels of father-reported familism compared to youth with lower levels of father-reported familism (*γ*_03_ = 0.52, 95% CI = [0.09, 0.99]), contrary to our hypotheses. At the cross-level, mother-reported familism significantly moderated the *within-youth* relationship between youth racial-ethnic discrimination and youth anger. Specifically, our results indicated that the *within-youth* association between youth racial-ethnic discrimination and youth anger was stronger for youth with higher levels of mother-reported familism compared to youth with lower levels of mother-reported familism (*γ*_11_ = 0.19; 95% CI = [0.05, 0.32]), contrary to our hypotheses. No other significant moderating effects at the cross-level were observed. In Panel B of Figure 1, we provide a graphical representation of the significant cross-level moderation effect for mother-reported familism. In this case, we plotted the *within-youth* effect of racial-ethnic discrimination on youth anger for different values of mother-reported familism: low (1 SD below the mean), average, and high (1 SD above the mean). As shown in Panel B of Figure 1, indeed, the *within-youth* association between youth racial-ethnic discrimination and youth anger was stronger for youth with high levels of mother-reported familism (1 SD above the mean) compared to youth with low levels of mother-reported familism (1 SD below the mean).

## Discussion

Overall, the current study contributes significantly to the literature by evaluating how family cultural values and processes may moderate daily associations between experiences of discrimination and distress among Mexican-origin youth. In particular, we examined the roles of familism, family cohesion, and ethnic-racial socialization, from the youth’s, mother’s, and father’s perspectives as potential protective factors that may buffer or attenuate anticipated daily associations between discrimination with negative affect and anger. Using a methodology that integrated micro-time (daily diary) and macro-time (Wave 1 baseline assessment in the longitudinal survey) research design features, we could examine daily differences between individuals and over time. We also examined variables at multiple ecological levels (individual-level, family-level protective factors) and multiple levels of analysis (between and within-person; between-level and cross-level moderation effects). These methodological innovations advance the current science on health disparities by allowing us to understand how moderating processes influence daily-level adolescent responses to discrimination-related stressors.

### Familism

First, focusing on familism, we found that youth-reported familism values significantly buffered daily associations between discrimination and negative affect and anger at the between-person level, as hypothesized. This finding is consistent with the prior research literature demonstrating robust macro-level associations between familism and youth well-being (e.g., Cahill et al., 2021). It extends this work to establish the protective effects of familism in moderating micro-level, daily associations between discrimination and negative affect for Mexican-origin youth. In contrast, however, father-reported familism values exacerbated daily associations between youth discrimination and indicators of distress (negative affect and anger) at the between-person level, and mother-reported familism exacerbated associations between discrimination and anger at the cross-level. The between-level interaction demonstrates that the adverse *between-youth* effect of racial-ethnic discrimination on negative affect was stronger for youth with higher levels of father-reported familism compared to youth with lower levels of father-reported familism. The cross-level interaction demonstrates that the day-to-day association between racial-ethnic discrimination and youth anger is stronger for youth with higher levels of mother-reported familism than for youth with lower levels of mother-reported familism. Familism, as a cultural value, emphasizes the importance of the family and encompasses several domains, including receiving support from family as well as obligation to the family (Sabogal et al., 1987). It is possible that youth’s emphasis on aspects of supportive familism explains the positive buffer within the youth model, as it has been found with Mexican-origin youth previously (Zeiders et al., 2013) whereas fathers may have emphasized obligative familism in surveys and in practice. Obligative familism, in contrast to supportive familism, has been found to be detrimental to mental health (see Valdivieso-Mora et al., 2016, for a review; Zeiders et al., 2013). Indeed, the obligation-related items on the measure of familism we used in the current study emphasized obligations that may have been more relevant and burdensome for adults than children (e.g., “A person should share his/ her home with uncles, aunts, or first cousins if they are in need” and “Aging parents should live with their relatives”). As such, youth may have felt relatively protected from the stress of fulfilling these obligations by their family at this point in their development, compared to their parents. Future research considering additional obligative familism items that are salient during adolescence (eg., responsibility for household chores, translating for parents, etc.) may be useful additions to further unpack these results. Although these exacerbating effects were not hypothesized, our results are consistent with prior literature reporting the negative effects of parent-reported familism (Delgado et al., 2011; Padilla et al., 2020). In particular, mother-reported familism has been found to exacerbate associations between discrimination and Mexican-origin adolescent adjustment, but for girls only (Delgado et al., 2011). Our findings are also similar to those of Padilla and colleagues, where youth and parent-reported familism had opposite directions of effect; father-reported familism was related to more youth-father conflict, whereas youth-reported familism was positively associated with positive parent-youth relationship quality in Mexican-origin families (Padilla et al., 2020).

Youth and parent perspectives on familism may be unique and largely unshared (Padilla et al., 2020). In the current study, youth report of familism was not significantly correlated with either mother- or father-reported familism. In contrast, the two parental reports were significantly positively associated with each other. Importantly, research on individual and shared family perspectives of familism values among Mexican-origin families has revealed that familism is best represented as an individual-level construct rather than a shared family perspective (Padilla et al., 2020). In addition, prior work evaluating the relative contributions of youth, parent, and shared perspectives of familism on positive family outcomes (such as parent-child relationship quality) demonstrates that the link between familism values and parent-youth relationship quality is driven almost exclusively by youth’s unique perspectives (Padilla et al., 2020). Together with our results, these findings suggest that youth’s own assessment of familism values may be a protective factor for Mexican-origin youth.

In contrast to the protective effects of youth perspectives on families, parents’ reports of familism exacerbated the youth discrimination to distress link. In our sample, we observed large discrepancies in how familism is experienced across generations, consistent with prior work (e.g., Telzer, 2010). These differences may be rooted in the classic acculturation gap-distress theory (Szapocznik & Kurtines, 1993), which posits that immigrant parents and their children experience differences in acculturation to and adherence to the cultural values of their countries of origin and country of residence. This gap leads to intergenerational cultural conflict and, in turn, greater distress for both youth and parents (e.g., Lui, 2015; Portes & Rumbaut, 2006; Szapocznik & Kurtines, 1993; Telzer, 2010). Future work should consider how cultural conflict associated with differences in familism values may explain the exacerbating effects of parental familism values on the discrimination to distress link. Alternately, when youth witness parents’ experiences of discrimination, they may empathize with the parents and experience distress. This may be especially true if parents are not receptive to discussing their own experiences of discrimination with their youth and/or model avoidance. Further research is necessary to unpack how mother and father perceptions of familism may interact with youth familism values to affect how discrimination affects youth distress at the daily level and well-being over time, and to disentangle how parent familism values may differentially moderate youth experiences of interpersonal discrimination versus vicarious parent discrimination.

### Family cohesion

Second, we evaluated family cohesion as a central family process that may be protective for youth coping with discrimination. Results based on youth-reported family cohesion supported our hypothesis: we observed a between-level interaction of family cohesion and daily discrimination on youth negative affect. The adverse *between-youth* effect of racial-ethnic discrimination on negative affect was weaker for youth with higher levels of self-reported family cohesion compared to youth with lower levels of self-reported family cohesion. As such, youth perceptions of family cohesion appear to help manage daily stress associated with discrimination. This finding is consistent with prior research on the direct positive effects of family cohesion on psychological distress (Rivera et al., 2008) and the positive associations between daily levels of cohesion and well-being indicators (Fosco & Lyden-Staley, 2020). Our work provides further evidence, beyond direct effects, to suggest that family cohesion, at least from the youth perspective, is protective in that it buffers associations between discrimination and distress for Mexican-origin youth.

In contrast, however, we found that mother-reported family cohesion *exacerbated* associations between discrimination and negative affect, and father-reported family cohesion was not a significant moderator. Like our explanation of diverging patterns of familism values between parents and youth, perhaps differences in perceptions of family cohesion between immigrant parents and their children lead to intergenerational cultural conflict that exacerbates associations between discrimination and distress. Indeed, higher family cohesion in the presence of family cultural conflict has been associated with higher youth psychological distress across diverse Latinx groups (Rivera et al., 2008). Further research with even larger samples will be necessary to unpack these results and evaluate a possible 3-way interaction between discrimination, youth-, and mother-reported family cohesion on youth distress. Another important research direction may be to capture daily assessments of family cohesion rather than dispositional or macro-level family cohesion, as recent research suggests this construct varies at the daily level and affects youth’s well-being and distress (Fosco & Lyden-Stayley, 2020).

### Ethnic-racial socialization

Turning to the ethnic-racial socialization results, we found two significant between-level moderating effects in the link between adolescents’ daily exposure to racial-ethnic discrimination and adolescents’ daily negative affect. Contrary to our hypotheses, cultural socialization (father’s report) and preparation for bias (father’s report) rather than promotion of mistrust *exacerbated* the association between adolescents’ daily exposure to discrimination and adolescents’ daily negative affect. These results counter the notion that cultural socialization and preparation for bias are socialization strategies that should help adolescents experience less psychological distress in the face of racial-ethnic discrimination (Hughes et al., 2006; Wang et al., 2020). The present results are consistent with one previous daily diary study that found that family ethnic-racial socialization was a risk factor, increasing the association between daily stress and the use of disengagement) as a coping strategy among Latinx adolescents (Santiago et al., 2016). However, this prior study did not differentiate between the sub-domains of ethnic-racial socialization or collect data from multiple informants. It is also possible that some coping strategies are associated with distress, but are still necessary as strategies to safely navigate hostile environments and discriminatory experiences. Broadly, our results are also consistent wth prior research on ethnic-racial socialization among undocumented Latinx parents that has been linked to youth depressive symptoms (Cross et al., 2020); however, in that work parents’ promotion of mistrust was associated with increased youth internalizing wherease we found that preparation for bias exacerbated youth negative affect.

As reported by Mexican-origin fathers, cultural socialization efforts and preparation for bias appear to be risk factors in the between-level association between their adolescent’s daily - exposure to racial-ethnic discrimination and their daily levels of negative affect. In traditional Mexican culture, fathers tend to be the authority figure in the family and have greater exposure to the world outside of the family (Cauce & Domenech-Rodriguez, 2002; Umaña-Taylor & Guimond, 2010). Fathers may also take on more parental involvement than historically assumed, and fathering may uniquely influence their children’s social and developmental outcomes (Cabrera & Bradley, 2012). Thus, fathers’ ethnic-racial socialization messages may carry particular weight and legitimacy.

On the one hand, because cultural socialization is geared towards instilling a sense of pride in one’s cultural heritage, assaults against one’s racial-ethnic identity in the form of racial-ethnic discrimination may feel subjectively amplified for those adolescents who have received cultural socialization messages from their fathers. In other words, greater cultural socialization may accentuate the sense of injustice that adolescents feel when exposed to racial-ethnic discrimination and stir up emotions such as anger, fear, or shame, strengthening the association between discrimination and negative affect. On the other hand, fathers’ preparation for bias may prime their adolescent children to be much more aware of the threat of racism and racial-ethnic discrimination in their ecological context. As a result, being the target (either directly, vicariously, or online) of discrimination may be more closely associated with negative affect for those adolescents who have received more cultural socialization and preparation for bias messages from their fathers, compared to those adolescents who received less frequent cultural socialization and preparation for bias messages. The effects of parent ethnic-racial socialization messages may especially exacerbate associations between discrimination and youth distress when youth are vicariously exposed to parents’ experiences of discrimination and are upset about those for whom they care the most. At the same time, it is crucial to keep in mind that all other tests of the potential moderating effects of these ethnic-racial socialization strategies (i.e., cultural socialization, preparation for bias, and promotion of mistrust) in the discrimination-distress link yielded null findings both for negative affect and anger.

### Limitations

The present study leveraged a between- and within-persons design to evaluate family values and processes that may assist youth in coping with daily racism. However, our study has some limitations that warrant attention. First, although our study included dynamic daily reports of discrimination and indicators of youth distress, our analyses did not fully disentangle directionality among these same-day variables. Future research that samples multiple time points within a day may detail how discrimination-distress dynamics occur within the same day and unfold across days. At the same time, we must be mindful of participant burden and the feasibility of daily data collection among youth. Second, given the already complex nature of evaluating moderating pathways in the daily association between discrimination and youth distress, we could not examine higher-order interactions, such as how youth and parent reports of familism and family cohesion may interact with each other and with discrimination. For example, it may be that the exacerbating effect of maternal report of family cohesion on the association between discrimination and youth negative affect is conditional on youth endorsement of low family cohesion. Third, although we observed several significant moderating effects on associations between racial-ethnic discrimination and youth negative affect and anger, the confidence intervals around some of these significant effects were large; as such, the collection of additional data, either via a larger sample size or additional days of daily diary, may help more precisely estimate the magnitude of these effects. Fourth, it is important to acknowledge that the alphas for the promotion of mistrust subscales are relatively low; this is likely related to the subscale being limited to two items and is consistent with other published studies (e.g., Park et al., 2020, mother α = 0.38, father α = 0.50). Fifth, our hypotheses were formed a priori but were not formally preregistered. Sixth, our data were collected from families following the onset of the COVID-19 pandemic and the impact of the pandemic youth was not explored. Notably, in the years following the pandemic Latinx youth demonstrated disproportionate increases in internalizing symptoms compared to other ethnic groups, especially among Mexican-origin youth (Polo et al., 2023), and recent work has highlighted the promotive role of family resilience, or the belief that the family is capable of overcoming, as a salient aspect of the family that is related to familism and has been associated with fewer youth internalizing symptoms following the pandemic (Stein et al., 2023). Finally, although it is a strength of the current study that we could include father reports alongside youth and maternal reports, we could not obtain father reports from some families. This becomes an important limitation in a context where cultural values and family processes are associated with fathers’ nonresponse; however, we are unable to evaluate this possibility. Given the vital role of -father-reported cultural values and family processes in moderating the association between youth discrimination and distress, including more fathers’ perspectives would be valuable.

## Future directions

Identifying youth-reported familism and family cohesion as protective in reducing the daily discrimination to distress link, alongside identifying several parent-reported family values and processes that are exacerbating, raises important issues to address in future research. For example, in the current study, we examined youth, mother, and father reports of potential protective factors that were assessed once (e.g., macro-level data). However, new work highlights daily fluctuations in family cohesion (Fosco & Lyden-Stayley, 2020) and family ethnic-racial socialization practices (Wang et al., 2023) that may have important implications for our understanding of these results. Incorporating the measurement of these factors at the daily level may be valuable for further delineating patterns of risk and protection for daily-level associations between discrimination and distress. Moreover, family and peers may assume differential socialization roles in everyday life. For example, recent work indicates that peer cultural socialization is particularly promotive of adolescents’ ethnic-racial identity. In contrast, family ethnic-racial socialization is central to coping after youth have experienced discrimination on a given day (Wang et al., 2023). Thus, incorporating peer ethnic-racial socialization may be informative. Disentangling more nuanced uses of ethnic-racial socialization practices may also be fruitful (e.g., Coard et al., 2023). For example, proactive ethnic-racial socialization conversations may provide youth with greater psychological protection than reactive approaches (e.g., following exposure to a discrimination experience; Derlan & Umaña-Taylor, 2015; Stein et al., 2021; Thomas, et al., 2009). In the context of this study, our self-report of ethnic-racial socialization does not provide details about when families are engaged in these processes. Therefore, future work collecting daily-level data on ethnic-racial socialization practices would enhance our understanding of these dynamics. Similarly, there may be important differences in the quality of how families engage in these processes beyond the quantity of ethnic-racial socialization practices. Planned future work examining observational assessments of family discussion of discrimination experiences with this sample may shed light on critical new dimensions of ethnic-racial socialization quality.

Additionally, our daily measure of discrimination asked about multiple sources of possible discrimination (any, online, vicarious, etc.). Variations in exposure to discrimination may be related to these differing sources of discrimination. Future research may seek to disentangle further effects of different sources of discrimination on frequency and intensity and youth mental health. Finally, it will be essential to determine how these daily indicators of distress are related to youth psychopathology and well-being. Planned future research using longitudinal data with this sample will allow us to evaluate how these micro-level associations and dynamics relate to macro-level assessment of psychopathology. Moreover, acknowledging that we collected these data with families in the two years following the onset of the COVID-19 pandemic, longitudinal data will allow us to understand more about the generalizability of our findings beyond the context of that public health crisis.

### Clinical implications

Overall, our findings have important practical implications, as recent research suggests that mechanisms of change identified by daily associations may be especially effective at improving individual outcomes in everyday life (Snippe et al., 2016). In particular, our results have implications for clinical interventions or prevention programs supporting mental health and well-being among Mexican-origin youth. First, enhancing youth’s own perceptions of familism values and increasing family cohesion may be protective in assisting youth in coping with daily experiences of discrimination. It is essential for youth themselves to endorse familism values and to perceive their families as cohesive. In contrast, our results suggest that it may not be helpful (and possibly aversive) if parents report high levels of familism and cohesion that youth do not see or agree with. Thus, individual efforts to promote daily well-being and less distress may be most effective if they focus on the youth’s perspective of familism and family cohesion. At the family-level, efforts to improve family communication about familism values and perspectives on family cohesion may also be a fruitful strategy to assist parents in understanding their adolescent’s attitudes. Adolescence is an optimal time for delivering such efforts, as youth’s perspectives on familism may be malleable and open to revision as they continue to develop across adolescence (Padilla et al., 2016).

Concerning the implications of our ethnic-racial socialization results for clinical practice, it is essential to recognize that although youth- and father-reported cultural socialization and preparation for bias may exacerbate feelings of negative affect and anger in response to discrimination, these ethnic-racial socialization efforts are valuable and important for helping youth cope in an unjust world where they are marginalized (Anderson & Stevenson, 2019). Indeed, feeling distressed following experiences of discrimination is undoubtedly valid, and further research is necessary to understand the adaptive or maladaptive nature of negative affect and anger responses to discrimination among Mexican-origin youth. Rather than targeting changes in ethnic-racial socialization practices or youth distress in response to discrimination, another approach to assisting youth in coping with racism could be integrating elements of Acceptance and Commitment Therapy (Hayes et al., 1999) into clinical practice. This form of therapy may assist youth in accepting and validating their emotions (e.g., feeling negative affect and anger) without judgment and as appropriate responses when confronted with racial-ethnic discrimination and in providing youth with tools so these emotions do not prevent them from moving toward their goals. Of note, the current study assessed anger independent of negative affect, where the latter encompasses feelings such as shame, anxiety, and fear. Doing so allows for more specificity in targeting clinical interventions. For example, there were no cross-level moderation effects for the daily link between discrimination and negative affect, suggesting that that association did not vary by family-level cultural differences. On the other hand, the daily link between discrimination and anger was stronger in the context of high maternal familism, indicating a potential avenue for mitigating the negative mental health effects of discrimination. Finally, in addition to clinical efforts aimed at helping youth cope with racism at the individual or family levels, efforts must be deployed at broader structural and systems levels to address racism.

## Figures and Tables

**Table 1.** Prevalence of daily discrimination experiences on each of the 21 daily diary days across all youth (% of youth reporting “Somewhat of a problem today” or “Very much of a problem today”)

		Interpersonal score			Vicarious score		
	Total score	Total	Today, others treated me poorly because of my race/ethnicity	Today, others treated me poorly online because of my race/ethnicity	Total	Today, others treated my friends, family, peers, poorly because of their race/ethnicity	Today, other people were treated poorly because of their race/ethnicity
Day	No. (%)	No. (%)	No. (%)	No. (%)	No. (%)	No. (%)	No. (%)
Day 1	45 (19.5%)	16 (6.9%)	11 (4.8%)	7 (3.0%)	38 (16.5%)	9 (3.9%)	35 (15.2%)
Day 2	35 (15.3%)	11 (4.8%)	8 (3.5%)	3 (1.3%)	33 (14.4%)	9 (3.9%)	29 (12.7%)
Day 3	26 (10.9%)	11 (4.6%)	9 (3.8%)	3 (1.3%)	22 (9.2%)	11 (4.6%)	20 (8.4%)
Day 4	17 (7.4%)	6 (2.6%)	5 (2.2%)	2 (0.9%)	14 (6.1%)	7 (3.0%)	13 (5.7%)
Day 5	15 (6.3%)	6 (2.5%)	5 (2.1%)	1 (0.4%)	12 (5.0%)	6 (2.5%)	10 (4.2%)
Day 6	27 (10.9%)	8 (3.2%)	6 (2.4%)	5 (2.0%)	25 (10.1%)	14 (5.7%)	21 (8.5%)
Day 7	24 (9.5%)	10 (4.0%)	8 (3.2%)	3 (1.2%)	18 (7.1%)	8 (3.2%)	13 (5.2%)
Day 8	23 (9.3%)	11 (4.5%)	11 (4.5%)	3 (1.2%)	19 (7.7%)	7 (2.8%)	17 (6.9%)
Day 9	26 (11.1%)	13 (5.6%)	11 (4.7%)	4 (1.7%)	22 (9.4%)	11 (4.7%)	17 (7.3%)
Day 10	17 (7.4%)	7 (3.0%)	4 (1.7%)	3 (1.3%)	14 (6.1%)	7 (3.0%)	10 (4.3%)
Day 11	18 (7.9%)	7 (3.1%)	6 (2.6%)	2 (0.9%)	16 (7.0%)	5 (2.2%)	14 (6.1%)
Day 12	15 (6.3%)	6 (2.5%)	3 (1.3%)	5 (2.1%)	10 (4.2%)	3 (1.3%)	9 (3.8%)
Day 13	18 (7.3%)	5 (2.0%)	3 (1.2%)	2 (0.8%)	15 (6.1%)	6 (2.4%)	14 (5.7%)
Day 14	18 (7.7%)	8 (3.4%)	7 (3.0%)	1 (0.4%)	14 (6.0%)	7 (3.0%)	11 (4.7%)
Day 15	20 (8.4%)	6 (2.5%)	5 (2.1%)	2 (0.8%)	17 (7.1%)	8 (3.3%)	13 (5.4%)
Day 16	15 (6.5%)	4 (1.7%)	4 (1.7%)	1 (0.4%)	15 (6.5%)	7 (3.0%)	13 (5.6%)
Day 17	12 (5.7%)	3 (1.4%)	3 (1.4%)	0 (0.0%)	12 (5.7%)	8 (3.8%)	8 (3.8%)
Day 18	13 (6.2%)	6 (2.8%)	6 (2.8%)	0 (0.0%)	11 (5.2%)	6 (2.8%)	7 (3.3%)
Day 19	11 (4.8%)	4 (1.8%)	3 (1.3%)	2 (0.9%)	9 (4.0%)	3 (1.3%)	7 (3.1%)
Day 20	15 (7.2%)	9 (4.3%)	8 (3.8%)	3 (1.4%)	11 (5.3%)	6 (2.9%)	9 (4.3%)
Day 21	14 (6.8%)	4 (2.0%)	4 (2.0%)	1 (0.5%)	12 (5.9%)	8 (3.9%)	8 (3.9%)
Average	424 (8.7%)	161 (3.3%)	130 (2.7%)	53 (1.1%)	359 (7.4%)	156 (3.2%)	298 (6.1%)

**Table 2.** Correlation between youth, mother, and father self-reports of the focal predictor, outcomes, and moderators

Variable	1	2	3	4	5	6	7	8	9	10	11	12	13	14	15	16	17	18
1. Daily racial-ethnic discrimination (youth report)^a^	1.00																	
2. Daily negative affect (youth report)^a^	0.30*	1.00																
3. Daily anger (youth report)^a^	0.31*	0.73*	1.00															
**Familism**																		
4. Youth report	−0.05	−0.09	−0.12*	1.00														
5. Mother report	0.00	−0.08	−0.08	0.11	1.00													
6. Father report	−0.13	−0.06	−0.04	0.10	0.34*	1.00												
**Family Cohesion**																		
7. Youth report	−0.18*	−0.12*	−0.15*	0.16*	−0.03	0.07	1.00											
8. Mother report	−0.03	0.02	−0.04	0.01	−0.01	0.08	0.15*	1.00										
9. Father report	−0.04	−0.07	−0.10	0.10	0.02	0.14	0.09	0.28*	1.00									
**Cultural Socialization**																		
10. Youth report	0.01	0.04	−0.04	0.17*	−0.08	−0.01	0.15*	0.15*	0.06	1.00								
11. Mother report	0.01	0.04	−0.02	0.14*	0.07	−0.06	0.00	0.05	−0.02	0.15*	1.00							
12. Father report	−0.08	−0.14	−0.02	0.08	0.04	0.08	0.03	0.11	0.23*	0.09	0.25*	1.00						
**Preparation for Bias**																		
13. Youth report	0.19*	0.14*	0.11	−0.02	0.01	0.04	0.00	0.09	0.07	0.55*	0.11	0.10	1.00					
14. Mother report	0.12*	0.02	−0.02	0.15*	0.15*	0.09	−0.04	−0.05	−0.06	0.07	0.53*	0.09	0.16*	1.00				
15. Father report	−0.08	−0.13	−0.05	0.11	0.04	0.07	0.04	0.06	0.11	0.13	0.13	0.72*	0.15	0.17*	1.00			
**Promotion of Mistrust**																		
16. Youth report	0.14*	0.15*	0.06	−0.04	0.05	0.10	−0.11*	−0.08	−0.13	0.24*	−0.02	−0.07	0.34*	0.06	−0.09	1.00		
17. Mother report	0.04	0.09	0.09	−0.03	0.13*	0.05	−0.11	−0.02	−0.08	−0.05	0.32*	0.05	0.00	0.42*	0.07	0.01	1.00	
18. Father report	−0.04	−0.05	−0.04	0.05	0.11	0.18*	−0.10	−0.09	−0.07	0.01	0.08	0.42*	−0.04	−0.01	0.53*	−0.10	0.23*	1.00
*Mean*	*4.2*	*7.4*	*43.6*	*20.1*	*18.9*	*20.7*	*27.1*	*29.4*	*29.1*	*2.7*	*2.9*	*2.6*	*2.0*	*2.5*	*2.3*	*1.4*	*1.3*	*1.5*
(SD)	(0.5)	(2.5)	(6.9)	(3.9)	(4.7)	(5.0)	(5.4)	(4.0)	(4.4)	(0.9)	(1.0)	(1.1)	(0.8)	(1.0)	(0.9)	(0.7)	(0.7)	(0.9)

Notes: SD = standard deviation.

a *Between-youth* correlations of the average daily levels of racial-ethnic discrimination, negative affect, and anger.

* *p* < 0.05.

**Table 3.** Moderating effects of youth-, mother-, and father-reported ethnic-racial socialization, familism, and family cohesion in the relationship between youth racial-ethnic discrimination and youth negative affect

	Youth Report	Mother Report	Father Report
Estimates	Coeff. [95% CI]	Coeff. [95% CI]	Coeff. [95% CI]
**Moderator: Familism**			
Moderation effects			
Between-level (γ_03_)	−0.26 [−0.45, −0.06]*	0.06 [−0.05, 0.16]	0.23 [0.08, 0.38]*
Cross-level (γ_11_)	0.03 [−0.02, 0.09]	0.04 [0.00, 0.08]	0.00 [−0.05, 0.05]
**Moderator: Family Cohesion**			
Moderation effects			
Between-level (γ_03_)	−0.09 [−0.18, −0.01]*	0.13 [0.01, 0.24]*	−0.11 [−0.36, 0.13]
Cross-level (γ_11_)	−0.01 [−0.05, 0.03]	−0.04 [−0.10, 0.03]	−0.01 [−0.06, 0.04]
**Moderator: Cultural Socialization**			
Moderation effects			
Between-level (γ_03_)	0.25 [−0.32, 0.82]	−0.21 [−0.92, 0.50]	0.83 [0.09, 1.57]*
Cross-level (γ_11_)	−0.03 [−0.21, 0.15]	0.11 [−0.10, 0.32]	−0.01 [−0.30, 0.27]
**Moderator: Preparation for Bias**			
Moderation effects			
Between-level (γ_03_)	0.62 [−0.004, 1.25]	0.13 [−0.47, 0.72]	1.23 [0.28, 2.18]*
Cross-level (γ_11_)	−0.01 [−0.21, 0.19]	0.13 [−0.08, 0.33]	0.16 [−0.13, 0.46]
**Moderator: Promotion of Mistrust**			
Moderation effects			
Between-level (γ_03_)	0.13 [−0.56, 0.81]	0.58 [−0.29, 1.45]	−0.07 [−1.75, 1.62]
Cross-level (γ_11_)	−0.07 [−0.30, 0.16]	−0.03 [−0.29, 0.22]	0.18 [−0.15, 0.51]

Notes. Coeff. = beta coefficient; CI = confidence interval.

* *p* < 0.05.

**Table 4.** Moderating effects of youth-, mother-, and father-reported ethnic-racial socialization, familism, and family cohesion in the relationship between youth racial-ethnic discrimination and youth anger

	Youth Report	Mother Report	Father Report
Estimates	Coeff. [95% CI]	Coeff. [95% CI]	Coeff. [95% CI]
**Moderator: Familism**			
Moderation effects			
Between-level (γ_03_)	−0.62 [−1.18, −0.06]*	0.23 [−0.06, 0.51]	0.52 [0.05, 0.99]*
Cross-level (γ_11_)	0.15 [−0.03, 0.32]	0.19 [0.05, 0.32]*	0.01 [−0.21, 0.22]
**Moderator: Family Cohesion**			
Moderation effects			
Between-level (γ_03_)	−0.03 [−0.26, 0.20]	0.02 [−0.35, 0.39]	0.03 [−0.46, 0.52]
Cross-level (γ_11_)	−0.02 [−0.15, 0.11]	−0.13 [−0.34, 0.08]	−0.04 [−0.24, 0.15]
**Moderator: Cultural Socialization**			
Moderation effects			
Between-level (γ_03_)	0.53 [−1.04, 2.11]	−0.31 [−2.28, 1.66]	2.39 [−0.10, 4.88]
Cross-level (γ_11_)	−0.06 [−0.65, 0.53]	0.15 [−0.51, 0.81]	0.18 [−0.64, 1.00]
**Moderator: Preparation for Bias**			
Moderation effects			
Between-level (γ_03_)	0.82 [−0.91, 2.56]	0.84 [−0.86, 2.55]	2.22 [−0.46, 4.90]
Cross-level (γ_11_)	0.06 [−0.57, 0.68]	0.28 [−0.38, 0.94]	0.85 [−0.12, 1.81]
**Moderator: Promotion of Mistrust**			
Moderation effects			
Between-level (γ_03_)	−0.66 [−2.55, 1.23]	1.02 [−1.33, 3.37]	−0.06 [−3.23, 3.12]
Cross-level (γ_11_)	−0.26 [−1.02, 0.49]	−0.30 [−1.12, 0.53]	0.92 [−0.31, 2.14]

Notes. Coeff. = beta coefficient; CI = confidence interval.

* *p* < 0.05.
